# Retrograde approach for recanalization in stumpless chronic total occlusion: A case report

**DOI:** 10.1002/ccr3.9504

**Published:** 2024-10-28

**Authors:** Dayu Wang, Ruibin Wei, Junteng Zheng, Zhen Liu, Jian Hou, Jianhao Li

**Affiliations:** ^1^ Department of Cardiology The Affiliated Panyu Central Hospital, Guangzhou Medical University Guangzhou China

**Keywords:** follow‐up studies, percutaneous coronary intervention, retrograde approach, revascularization, Stumpless chronic total occlusion

## Abstract

Chronic total occlusive disease of the coronary arteries is the most challenging disease in the field of coronary intervention. When the anterograde approach is not feasible, the retrograde approach remains the only strategy.

## 1 INTRODUCTION

Chronic total occlusion (CTO) disease is a complex form of coronary artery disease that poses a technical challenge in percutaneous coronary intervention (PCI).^1^ Despite these challenges, CTO‐PCI has emerged as an effective treatment strategy, substantially enhancing the patient's quality of life.^2^, ^3^, ^4^ Thus, understanding the various technologies employed in CTO‐PCI is crucial to optimize the outcomes.

The two primary technologies utilized in CTO‐PCI are the forward and reverse approaches, each with a distinct methodology. Forward techniques include guided wire passage and dissection re‐entry into the true cavity, while reverse methods involve similar maneuvers in a retrograde fashion.^5^, ^6^ The antegrade approach is typically the preferred strategy for CTO revascularization due to its direct accessibility. However, the antegrade approach can sometimes be impractical or unsuccessful, particularly in cases of complex lesion anatomy, heavy calcification, or unfavorable entry points, where the guidewire cannot navigate the occlusion effectively.

In such scenarios, the retrograde approach becomes indispensable. The retrograde approach involves accessing the occluded segment from a distal collateral vessel or a secondary coronary artery, allowing for alternative entry points that may be more favorable for wire crossing.^7^, ^8^ This method is crucial when the antegrade approach fails because it provides additional pathways and strategies to navigate the occlusion, leveraging the natural collateral circulation to reach the distal cap of the CTO.^9^, ^10^ Moreover, the retrograde approach can facilitate the visualization and manipulation of the guidewire from different angles, increasing the likelihood of successful recanalization.^11^, ^12^

Herein, we demonstrate the retrograde approach for the recanalization of a stumpless CTO lesion. By elucidating the rationale for selecting the retrograde approach in scenarios where antegrade methods are not feasible, we aim to provide a clearer understanding of its significance in the context of CTO‐PCI. Moreover, we aim to underscore the importance of strategic decision‐making in navigating the intricacies of CTO management, ultimately contributing to improved patient outcomes.

## 2 CASE HISTORY

A 67‐year‐old man presented to our hospital with chest tightness and shortness of breath for the past 3 h. He had a significant medical history of hypertension, diabetes mellitus, hyperlipidemia, and renal insufficiency. Six months prior, he had undergone stent insertion for severe stenosis in the left internal carotid artery, with no residual sequelae.

Upon admission, the patient exhibited Grade 3 hypertension and symptoms suggestive of unstable angina. Electrocardiogram findings revealed left ventricular hypertrophy and ST‐T changes in leads V4‐V6. Echocardiogram demonstrated a left ventricular ejection fraction <58%. Laboratory investigations indicated elevated levels of amino‐terminal B‐type natriuretic peptide, troponin, creatinine, and urea, suggesting cardiac and renal dysfunction. The preliminary diagnosis was heart failure.

The patient received antiplatelet, lipid‐lowering, gastric‐protective, antihypertensive, renal‐protective, and anti‐heart failure treatments.

### 2.1 Coronary angiography findings

To further investigate the decline in cardiac function, coronary angiography was performed on the sixth day after admission. The patient's SYNTAX score was calculated to be 17.5.

1. *Proximal‐mid anterior descending artery*: Diffuse stenosis of 60%–70%.
2. *Collateral circulation*: From the anterior descending branch to the mid‐right coronary artery via Grade 2 collateral connections (CC).
3. *Proximal‐mid left circumflex branch*: Diffuse stenosis of 80%–90%.
4. *Right coronary artery* (*RCA*): Presence of CTO.

**FIGURE 1:**
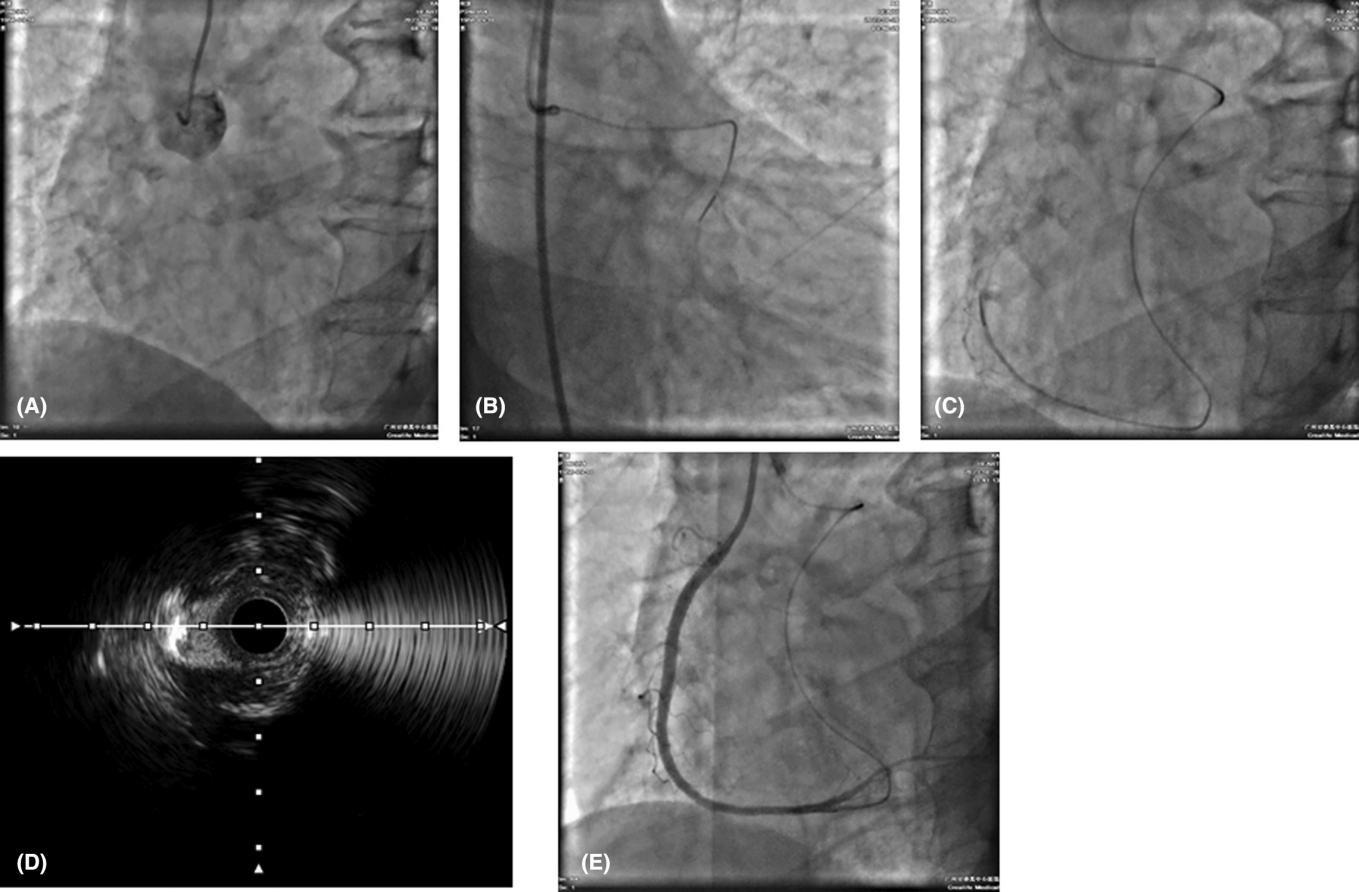
Multiple examination images. (A) Stumpless chronic total occlusion (CTO). (B) Retrograde approach is established via Grade 2 collateral connection. (C) SUOH03 guidewire advances through Grade 2 collateral connection to cross the occlusion at the right coronary artery (RCA) opening to reach the ascending aorta. (D) Intravascular ultrasound (IVUS) findings indicating CTO. (E) Dilation of the obstructed vessel.

Given these findings, PCI was recommended to alleviate the obstruction (Figure 1A and Movie 1).

**MOVIE 1:** Stumpless chronic total occlusion.

## 3 METHODS

Evaluation on the CTO entry showed that the patient had a stumpless CTO lesion without clear entry, precluding the use of the antegrade approach. Hence, collateral circulation (Grade 2 CC) was formed via the retrograde approach to cross the occlusion at the RCA opening to reach the ascending aorta, and active pulling was performed to externalize the wire and finally perform antegrade intervention (Figure 1B–D and Movie 2,3).

**MOVIE 2:** Retrograde approach is established via Grade 2 collateral connection.

**MOVIE 3:** SUOH03 guidewire advances through Grade 2 collateral connection to cross the occlusion at the right coronary artery (RCA) opening to reach the ascending aorta.

Briefly, the procedure began with a 2.6F, 130 cm microcatheter to guide an ASAHI SUOH 03 wire through the collateral circulation to the midsection of the RCA. The microcatheter was then advanced, and the wire was exchanged for an ASAHI Fielder XT‐A, which crossed the fibrous cap at the right coronary ostium and extended into the ascending aorta. After advancing the microcatheter further, the wire was replaced with an ASAHI RG3, which also reached the ascending aorta. An antegrade 6F JR4 guiding catheter equipped with a snare was employed to capture and externalize the ASAHI RG3 guidewire. This was followed by an antegrade intravascular ultrasound (IVUS) with the ASAHI RG3 wire to measure the lumen diameter of the RCA, culminating in the stent placement.

The therapeutic regimen for this CTO patient included secondary prevention medications for coronary artery disease: aspirin, clopidogrel, and atorvastatin. Given the patient's compromised renal function, felodipine was selected for blood pressure control.

## 4 POTENTIAL RISKS AND COMPLICATIONS

Several potential risks and complications were considered during the procedure. Firstly, the retrograde approach and manipulation of wires through collateral channels increased the risk of vessel perforation, which could lead to pericardial effusion or tamponade. Then, both the antegrade and retrograde maneuvers carried the risk of causing coronary dissection, potentially leading to acute vessel closure. Moreover, the process of externalizing the guidewire carried a risk of wire entrapment, which could complicate the procedure and necessitate additional interventions. Finally, given the patient's pre‐existing renal insufficiency, the use of contrast agents posed a risk of exacerbating renal dysfunction.

## 5 CONCLUSION AND RESULTS

The patient was discharged from the hospital on the fourth day after PCI. For the third month post‐Discharge, the physical examination showed that No recurrence of chest pain or dyspnea. The laboratory tests indicated the stable renal function with normal creatinine and urea levels. Throughout the follow‐up period, the patient adhered to the prescribed medication regimen, including antiplatelet, lipid‐lowering, antihypertensive, and renal‐protective agents. He also maintained a healthy lifestyle with regular physical activity and dietary modifications. The retrograde approach via collateral circulation proved to be a successful therapy for stumpless CTO revascularization, as evidenced by the patient's favorable recovery and absence of complications. This case suggests that such an approach can be considered for similar patients to achieve effective revascularization (Figure 1E and Movie 4). However, it is important to note that the retrograde approach has certain limitations and contraindications. Firstly, the procedure is technically demanding and requires a high level of operator skill and experience. Secondly, the success of the retrograde approach is highly dependent on the presence of adequate collateral circulation, which may not be available in all patients. Thirdly, potential complications include vessel perforation, collateral channel injury, and other serious adverse events. Therefore, careful assessment of the patient's condition and improved technique will help the clinical benefits of retrograde approaches outweigh their potential risks. This may provide a safer and more effective treatment option for stumpless CTO revascularization.

**MOVIE 4:** Dilation of the obstructed vessel.

## 6 DISCUSSION

### 6.1 Influence of anatomical characteristics on CTO‐PCI strategies

Proximal fibrous cap morphology, CTO lesion length, and anatomical characteristics (presence of tortuosity and calcification), occlusive distal vessel conditions, and collateral circulation conditions influence the development of CTO‐PCI strategies.^13^ These factors play a crucial role in determining the appropriate approach for revascularization. In our case, the absence of a visible stump and the unclear entry point posed a significant challenge for revascularization. The inability to use IVUS for guidance further complicated the procedure, underscoring the need for a retrograde approach based on the patient's specific anatomical conditions.

### 6.2 Guidelines and recommendations for retrograde approach

Different regional guidelines provide criteria for utilizing the retrograde approach in CTO‐PCI. The Asia‐Pacific CTO Club suggests using the retrograde approach of CTO‐PCI based on the following criteria: unclear proximal fibrous cap (IVUS cannot be used), poor condition of the distal blood vessels, and availability of collateral vessels.^14^ The Japanese CTO Club suggests using the retrograde approach for CTO‐PCI based on the criteria of previous failure, occlusion length ≥20 mm, no stump, and availability of collateral vessels.^13^ China's updated version of the CTO‐PCI pathway suggests using the retrograde approach based on the unclear display of the proximal fibrous cap, inability to use IVUS or failure of IVUS guidance, and availability of collateral vessels. However, in our case, the patient had a stumpless CTO lesion with unclear entry and available collateral vessels, and failure of IVUS guidance; thus, collateral circulation (Grade 2 CC) was established via the retrograde approach to cross the occlusion at the RCA opening to reach the ascending aorta. Moreover, active pulling was performed to externalize the wire and finally perform antegrade intervention (Figure 1B–D and Movie 2,3). Our data could provide a reference for stumpless CTO revascularization.

### 6.3 Challenges of stumpless CTO revascularization

Stumpless CTO, a specialized category of CTOs, is considered a persistent revascularization challenge. The entrance of the stumpless lesion is often located at the bifurcation; hence, it is difficult to locate the CTO entrance accurately, resulting in the guidewire often slipping into a side branch.^15^ Therefore, an IVUS catheter is often used to extend into the side branch of the CTO lesion by using the initially positioned wire and then drawing back until the IVUS can visualize the CTO cap's entrance point. In our case, the guidewire's tendency to slip into a side branch made the use of IVUS particularly critical. However, due to the failure of IVUS guidance in this patient, we had to rely on the retrograde approach as the primary strategy for revascularization.

### 6.4 Predictors for successful PCI approaches

Our patient presented stumpless CTO without forward blood flow and vascularization. Thus, a retrograde approach for recanalization was used as in previous studies.^16^, ^17^ Wu et al. showed that a J‐CTO score <2 and the presence of microchannels at the proximal stump were considered predictors for a successful antegrade‐only approach in PCI for ostial or stumpless CTO.^18^ Another study focusing on the antegrade procedure indicated that a stumpless lesion was an independent predictor of failed antegrade PCI, and a score of two points for the stumpless lesion improves the value of the current J‐CTO score in predicting the procedure outcome.^19^ Our experience aligns with findings that stumpless lesions are independent predictors of failed antegrade PCI, reinforcing the importance of a tailored approach based on specific lesion characteristics.

### 6.5 Variation in success rates

The varying proportions of cases undergoing the retrograde approach at different centers could lead to different levels of operators' skill and experience and great differences in the success rate of stumpless CTO‐PCI among different centers. Therefore, multicenter studies with large sample sizes are warranted for results close to real‐world conditions.

### 6.6 Limitations of the retrograde approach

The retrograde approach is an alternative to the antegrade approach; however, it has some limitations. First, the operation is difficult to popularize. Second, the availability of collateral vessels is limited. Third, there are many associated complications, such as coronary hematoma and perforation. When the retrograde approach is not viable, coronary artery bypass grafting is considered the primary treatment strategy.

## AUTHOR CONTRIBUTIONS

**Dayu Wang:** Conceptualization; data curation; formal analysis; funding acquisition; investigation; methodology; validation; visualization; writing – original draft; writing – review and editing. **Ruibin Wei:** Visualization. **Junteng Zheng:** Visualization. **Zhen Liu:** Visualization. **Jian Hou:** Conceptualization; data curation; formal analysis; investigation; writing – original draft; writing – review and editing. **Jianhao Li:** Conceptualization; data curation; formal analysis; investigation; methodology; validation; visualization; writing – original draft; writing – review and editing.

## FUNDING INFORMATION

This study was supported by the 2023 Hospital Fund of Guangzhou Panyu Central Hospital (Research Fund of the Institute, PY‐2023‐011) and the Medical Health Science and Technology Project of Panyu District (2021‐Z04‐082).

## CONFLICT OF INTEREST STATEMENT

The authors declare no conflicts of interest.

## ETHICS STATEMENT

Not required.

## CONSENT

Written informed consent was obtained from the patient to publish this report in accordance with the journal's patient consent policy.

## Data Availability

The data that support the findings of this study are available from the corresponding author upon reasonable request.
